# Estimating the infection and case fatality ratio for coronavirus disease (COVID-19) using age-adjusted data from the outbreak on the Diamond Princess cruise ship, February 2020

**DOI:** 10.2807/1560-7917.ES.2020.25.12.2000256

**Published:** 2020-03-26

**Authors:** Timothy W Russell, Joel Hellewell, Christopher I Jarvis, Kevin van Zandvoort, Sam Abbott, Ruwan Ratnayake, CMMID COVID-19 working group, Stefan Flasche, Rosalind M Eggo, W John Edmunds, Adam J Kucharski

**Affiliations:** 1 Centre for the Mathematical Modelling of Infectious Diseases, Department of Infectious Disease Epidemiology, London School of Hygiene and Tropical Medicine, London, United Kingdom; 2 These authors contributed equally to this work; 3 Department of Infectious Disease Epidemiology, London School of Hygiene and Tropical Medicine, London, United Kingdom; 4 The members of the Centre for the Mathematical Modelling of Infectious Diseases (CMMID) COVID-19 working group are listed at the end of the article

**Keywords:** Case fatality ratio, infection fatality ratio, COVID-19, outbreak, severity, asymptomatic, coronavirus, cruise ship

## Abstract

Adjusting for delay from confirmation to death, we estimated case and infection fatality ratios (CFR, IFR) for coronavirus disease (COVID-19) on the Diamond Princess ship as 2.6% (95% confidence interval (CI): 0.89–6.7) and 1.3% (95% CI: 0.38–3.6), respectively. Comparing deaths on board with expected deaths based on naive CFR estimates from China, we estimated CFR and IFR in China to be 1.2% (95% CI: 0.3–2.7) and 0.6% (95% CI: 0.2–1.3), respectively.

In real time, estimates of the case fatality ratio (CFR) and infection fatality ratio (IFR) can be biased upwards by under-reporting of cases and downwards by failure to account for the delay from confirmation to death. Collecting detailed epidemiological information from a closed population such as the quarantined Diamond Princess cruise ship in Japan can produce a more comprehensive description of asymptomatic and symptomatic cases and their subsequent outcomes. Our aim was to estimate the IFR and CFR of coronavirus disease (COVID-19) in China, using data from passengers of the Diamond Princess while correcting for delays between confirmation and death and for the age structure of the population. 

## Situation on the cruise ship

On 1 February 2020, a patient tested positive for COVID-19 in Hong Kong; they had disembarked from the Diamond Princess cruise ship on 25 January [1,2]. This patient had had onset of symptoms on 19 January, one day before boarding the ship [1]. After its return to Yokohama, Japan, on 3 February, the ship was held in quarantine, during which testing was performed in order to measure COVID-19 infections among the 3,711 passengers and crew members on board. 

Passengers of the Diamond Princess were initially to be held in quarantine for 14 days until 17 February. However, those who had intense exposure to the confirmed case-patient, such as sharing a cabin, were held in quarantine beyond the initial 14-day window [2]. According to reference [2], by 20 February, there were 619 confirmed cases on-board (17%), 318 of them were asymptomatic (asymptomatic cases were either self-assessed to be symptomless or tested positive before symptom onset) and 301 were symptomatic [2]. Overall 3,063 PCR tests were performed among passengers and crew members. Testing started among the elderly passengers, descending by age [2]. For details on the testing procedure, see [1] and [2].

## Adjusting for outcome delay in case fatality ratio estimates

To date, there have been many estimates of the CFR of COVID-19, far too many to exhaustively summarise here. However, to give an idea, estimates range from 0.4% (95% confidence interval (CI): 0.4–0.5) [3] to 3.8% [4]. During an outbreak, the so-called naive CFR (nCFR), i.e. the ratio of reported deaths date to reported cases to date, will underestimate the true CFR because the outcome (recovery or death) is not known for all cases [5,6], assuming all cases are detected. We can estimate the true denominator for the CFR (i.e. the number of cases with known outcomes) by accounting for the delay from confirmation to death [6]. We assumed that the delay from confirmation to death followed the same distribution as the estimated time from hospitalisation to death, based on data from the COVID-19 outbreak in Wuhan, China, between 17 December 2019 and 22 January 2020, accounting for underestimation in the data as a result of as-yet-unknown disease outcomes (Figure, panels A and B) [7]. As a sensitivity analysis, we also considered raw ‘non-truncated’ distributions, which do not account for censoring (i.e. because of the continued growth of the outbreak, cases with shorter incubation periods are more likely to be included in the data set); the raw and truncated distributions (Supplementary Figure S1) had a mean of 8.6 days and 13 days, respectively (Supplementary Tables S1 and S2).

**Figure:**
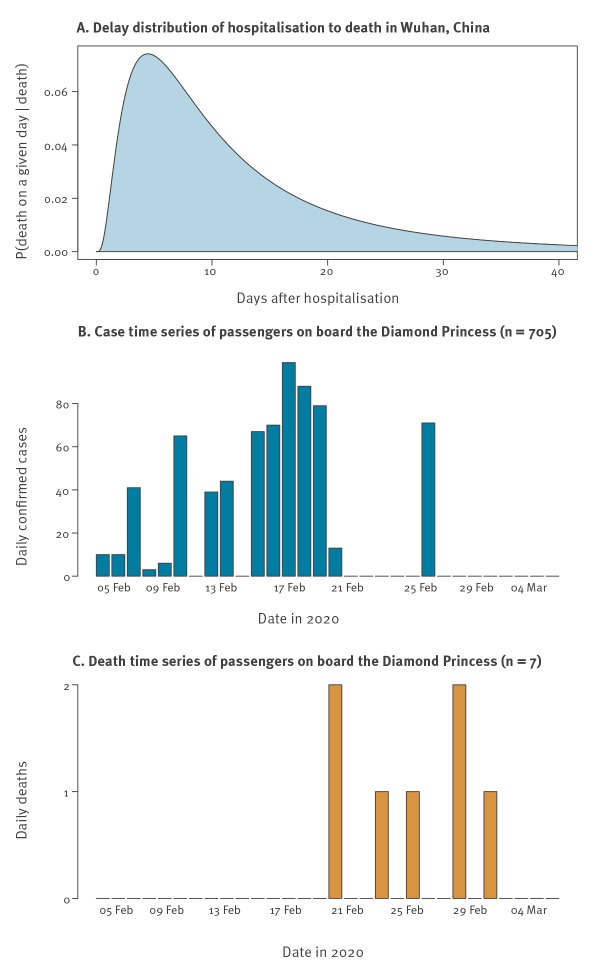
The time-to-death distributions and case and death data used to calculate the corrected case fatality estimates, Diamond Princess cruise ship, February 2020 (n = 3,711)

Panel A represents a lognormal distribution fitted and reported in Linton et al. [7] (Table 2) using data from the outbreak in Wuhan, China. The distribution has a mean of 13 days and standard deviation of 12.7 days. Case and death time-series data is as reported until 5 March 2020 and is taken from [8]. Nine negative cases on 5 March were removed from the figure for aesthetic purposes. However, they were included in the analysis.

## Data sources

We used data from two different sources in our analyses. Time-series for the date of confirmation of cases and the date of each death were taken from the World Health Organization situation reports [8], using data up to 5 March. The breakdown of which cases were symptomatic and which were asymptomatic was taken from [1] and [2], which included data up to 20 February. There were 634 cases in total by 20 February according to [8] and 696 by 5 March. The asymptomatic vs symptomatic breakdown was taken from a total of 619 cases [1]. For comparison, we ran an uncorrected version of the analysis using data up to 25 March in the Supplementary Material. Nine negative cases were officially reported on 5 March [8], which we included in our analysis but omitted from Figure 1 for aesthetic purposes.

To adjust the CFR to account for delay to outcome, we use the method developed in [6] where case and death incidence data are used to estimate the number of cases with known outcomes, i.e. cases where the resolution, death or recovery, is known to have occurred:

$$Formula (1):u_{t}=\;\frac{\sum_{i=0}^{t}\sum_{j=0}^{\infty }c_{i-j}f_{j}}{\sum_{i=0}^{t}c_{j}},$$

where *c_t_* is the daily case incidence at time *t*, (with time measured in calendar days), *f_t_* is the proportion of cases with delay *t* between onset or hospitalisation and death; *u_t_* represents the underestimation of the known outcomes [6,7] and is used to scale the value of the cumulative number of cases in the denominator in the calculation of the cCFR. Given that asymptomatic infections are typically not reported, especially during an ongoing outbreak of a novel infection, this correction is normally used to calculate the cCFR. However, because of the high level of testing on the cruise ship, we were able to use this correction to calculate the corrected IFR (cIFR). After that, we used the measured proportions of asymptomatic to symptomatic cases on the Diamond Princess to scale the cIFR to estimate the cCFR. Method and data are available at: https://github.com/thimotei/cCFRDiamondPrincess.

## Corrected infection fatality ratio and case fatality ratio estimates

We estimated that the all-age cIFR on the Diamond Princess was 1.3% (95% confidence interval (CI): 0.38–3.6) and the cCFR was 2.6% (95% CI: 0.89–6.7) (Table 1). However, as the age distribution on the ship was skewed towards older individuals (mean age: 58 years), we also report age-stratified estimates. Using the age distribution of cases and deaths on the ship [1,2] to estimate for only individuals 70 years and older, the cIFR was 6.4% (95% CI: 2.6–13) and the cCFR was 13% (95% CI: 5.2–26) (Table 1). The 95% CI were calculated with an exact binomial test, with death count and either cases or known outcomes (depending on whether it was an interval for the naive or corrected estimate).

**Table 1:** Corrected infection fatality ratio and corrected case fatality ratio estimates calculated from reported case and death data, Diamond Princess cruise ship, February 2020 (n = 696)

Age group	cIFR (95% CI)	cCFR (95% CI)
All ages combined	1.3% (0.38–3.6)	2.6% (0.89–6.7)
≥ 70 years	6.4% (2.6–13)	13% (5.2–26)

CI: confidence interval; cCFR: corrected case fatality ratio; cIFR: corrected infection fatality ratio.

Data source: [8]. Correction was performed using Formula (1) and the hospitalisation-to-death distribution in [9].

Using an approach similar to indirect standardisation [9], we used the age-stratified nCFR estimates reported in a large study in China [10] to calculate the expected number of deaths of people on board the ship in each age group, (assuming this nCFR estimate in the standard population was accurate). This produced a total of 15.15 expected deaths, which corresponds to a nCFR estimate of 5% (15.15/301) for the Diamond Princess (Table 2), which falls within the top end of our 95% CI. As our cCFR for Diamond Princess was 2.6% (95% CI: 0.89–6.7), this suggests we need to multiply the nCFR estimates in China [7] by a factor 52% (95% CI: 14–100) to obtain the correct value. As the raw overall nCFR reported in the data from China was 2.3% [10], this suggests the cCFR in China during that period was 1.2% (95% CI: 0.3–3.1) and the IFR was 0.6% (95% CI: 0.2–1.7). Based on cases and deaths reported in China up to 4 March 2020, the nCFR calculation was considerably higher than the cCFR we estimate here (based on data taken from [8], nCFR = 2,984/80,422 = 3.71% (95% CI: 3.58–3.84)). The confidence intervals calculated for China using an indirect standardisation method reflect the uncertainty in the Diamond Princess estimates, as it is carried forward in the scaling.

**Table 2:** Age-stratified cases, external nCFR estimates calculated during the outbreak in China, expected deaths on board the Diamond Princess using these nCFR estimates and the observed number of deaths, February 2020 (n = 619)

Age group (years)	Cases	External nCFR (95% CI)	Expected deaths using external nCFR (95% CI)	Observed deaths on cruise ship
0–9	0	0.0% (0.0–0.9)	0 (0–0)	0
10–19	2	0.2% (0.0–1.0)	0 (0–0)	0
20–29	25	0.2% (0.1–0.4)	0.05 (0.02–0.10)	0
30–39	27	0.2% (0.1–0.4)	0.06 (0.04–0.10)	0
40–49	19	0.4% (0.3–0.6)	0.08 (0.06–0.12)	0
50–59	28	1.3% (1.1–1.5)	0.36 (0.31–0.43)	0
60–69	76	3.6% (3.2–4.0)	2.74 (2.5–3.1)	0
70–79	95	8.0% (7.2–8.9)	7.6 (6.8–8.4)	3
80–89	29	14.8% (13.0–16.7)	4.28 (3.8–4.9)	4
**Total**	**301**	**NA **	**15.15 (13.5**–**17.1)**	**7**

cCFR: corrected case fatality ratio; CFR: case fatality ratio; CI: confidence interval; cIFR: corrected infection fatality ratio; NA: not applicable; nCFR: naïve case fatality ratio.

Data source: [2] for age-stratified data of cases with symptoms.

External nCFR refers to the CFR calculated during the outbreak in China [7]. Age-stratified case data are taken from [1,2]. The expected number of cases in each age group are calculated assuming that the nCFR estimates were correct on the ship, where the total number of expected deaths under these estimates was 15.15. Data on symptomatic/asymptomatic breakdown and the total number of cases by 20 February were taken from [2] (see Supplementary Table S2 for a more detailed version of this Table).

## Discussion

As at 24 March 2020, there have been 386,317 confirmed cases of coronavirus disease 2019 (COVID-19), with 16,713 deaths [8]. It is challenging to accurately estimate the CFR in real time [5,11], especially for an infection with attributes similar to COVID-19, which has a delay of almost 2 weeks between confirmation and death, strong effects of age and comorbidities on mortality risk, and likely under-reporting of cases in many settings [10]. Using an age-stratified adjustment method, we accounted for changes in known outcomes over time. By applying this method to data from the Diamond Princess, we focused on a setting that was likely to have lower reporting error because large numbers were tested and the test had high sensitivity. 

As the mean age on board the ship was 58 years, our cCFR estimates cannot directly be applied to a younger population; we therefore scaled our estimates to obtain values for a population with an age distribution equivalent to that in the outbreak in China. Although the Diamond Princess cohort was older, meaning that some deaths could be attributable to other causes, the natural death rate would have been much slower than the fatalities attributable to COVID-19. Given the limited background effect – and to ensure consistency with standard estimates of CFR rather than a hybrid ‘burden over natural rate’ calculation – we assumed that all deaths among COVID-19 cases were the result of COVID-19.

Our analysis had additional limitations. Cruise ship passengers may have a different health status to the general population of their home countries, owing to health requirements to embark on a multi-week holiday, or differences related to socioeconomic status or comborbities. Deaths only occurred in individuals 70 years or older, so we were not able to generate age-specific cCFRs; the fatality risk may also have been influenced by differences in healthcare between countries. Because of likely age-specific differences in reporting, we focused on overall cCFR in China, rather than calculating age-specific cCFRs [10,11]. In doing so, we were assuming that there were no age-specific differences in under-reporting. The main source of potential bias in this assumption is the age-specific severity level of COVID-19; there may be far lower levels of detection in children if their symptoms are milder, meaning that they are tested less often. 

## Conclusion

Our analysis shows the importance of adjusting for delays from confirmation to outcome in real-time estimates of fatality risk, and the benefits of combining datasets alongside appropriate age adjustments to provide early insights into COVID-19 severity.

## Centre for Mathematical Modelling of Infectious Disease 2019-nCoV working group

Thibaut Jombart, Amy Gimma, Nikos I Bosse, Alicia Rosello, Mark Jit, James D Munday, Billy J Quilty, Petra Klepac, Hamish Gibbs, Yang Liu, Sebastian Funk, Samuel Clifford, Fiona Sun, Kiesha Prem, Charlie Diamond, Nicholas Davies, Carl A B Pearson.

## Supplementary Data

205000 Supplement
