# Supplementary material for: Estimating the infection and case fatality ratio for coronavirus disease (COVID-19) using age-adjusted data from the outbreak on the Diamond Princess cruise ship, February 2020
Source: Euro Surveill. 2020 Mar 26;25(12):2000256. doi: 10.2807/1560-7917.ES.2020.25.12.2000256 (PMC7118348; doi:10.2807/1560-7917.ES.2020.25.12.2000256)
Supplement: Supplement [file 20-00256_RUSSEL_Supplement.pdf]

Estimating the infection and case fatality ratio for coronavirus disease (COVID-19) using age-adjusted data from the outbreak on the Diamond Princess cruise ship, February 2020

Supplementary Material

This supplementary material is hosted by *Eurosurveillance* as supporting information alongside the article "*Estimating the infection and case fatality ratio for coronavirus disease (COVID-19) using age-adjusted data from the outbreak on the Diamond Princess cruise ship, February 2020*", on behalf of the authors, who remain responsible for the accuracy and appropriateness of the content. The same standards for ethics, copyright, attributions and permissions as for the article apply. Supplements are not edited by *Eurosurveillance* and the journal is not responsible for the maintenance of any links or email addresses provided therein.

Detailed Methods

Sensitivity analysis

To account for the uncertainty in the fitted parameters of the hospitalisation-to-death delay distribution from Linton et al. [1], we calculate the CFR and IFR estimates with distributions parameterised using all four combinations of the minimum and maximum mean and SD reported. We then choose the smallest and largest CIs out of the four possible combinations as the resulting lower and upper CI respectively. We are able to do this rather than anything more sophisticated, as the relationship between the mean (and SD) of the delay distribution and the overall CFR or IFR estimate is very simple.

After performing the sensitivty analysis exercise, we find that the confidence intervals originally determined by a 95% exact binomial test (calculated using the number of cases and the "known outcomes" quantity derived in the correction) widen slightly at the top end only. We therefore report the wider CI interval and use it in any subsequent calculation.

Non-truncated distribution

When fitting the hospitalisation-to-death distribution to data, Linton et al. performed some analysis which accounted for right-truncation of the data [1]. This truncated distribution is most likely a more accurate estimate of the true distribution, which is why it was used in the analysis reported in the main text. However, for completeness, we present the difference in the two distributions here [Figure S1] and the effect the difference in distribution has on the results of the cCFR and cIFR calculated on the cruise ship [Table S1]. As was to be expected, a shorter mean delay produces fewer corrected for "known outcomes", meaning that the correction doesn't increase the naïve estimate by as much. Therefore, using the

truncated distribution (with a higher mean and standard deviation) in the calculation results in higher values for the cCFR and cIFR.

Indirect-standardisation

We standardise the age-stratified estimates for the CFR in China using what we believe to be an estimate of the effect the many biases present in such a value if it is estimated during an on-going outbreak. Arguably the largest such bias is the underreporting of cases, which is inevitable in a country with an overwhelmed healthcare. To this end, we treat the ratio between the CFR calculated based on the observed number of deaths on the Diamond Princess cruise ship and the expected number (if the nCFR in China had been true on the cruise ship) as the scaling factor by which we adjust the China data. In doing so, we are able to use all of the information in their age-stratified data with a high sample size along with the information in our CFR estimates calculated in a setting with no underreporting bias.

Limitations

The assumption that the delay between hospitalisation-to-death is equivalent to the delay between confirmation-to-death was undertaken as the data was reported by date of confirmation [1]. However, we implicitly test how sensitive our estimates are to this assumption by bootstrapping over the uncertainty range given in Linton et al. [1] and by calculating the estimates using both the truncated and non-truncated distributions. This is an inexact and indirect way to test the sensitivity of the estimates against this assumption. However, it is clear it makes very little difference (at the two significant figures level of precision reported) to the estimates.

More recent data

We performed the analysis based on the data at the time of 5th March, using primarily the data in [2, 3], as we required symptomatic/asymptomatic level data to estimate the IFR as well as the CFR. At this point, there has been a total of 7 deaths and 634 cases, of which ew have the breakdown of syptomatics to asyptomatics for 619 cases [2, 3]. Since there have been a further two deaths and 62 more cases. We are entering the phase where the correction does not need to correct for much, given that most outcomes are now known. This gives us a unique opportunity to test our corrected value against the naïve calculation, which after enough time should converge. We find that using the current available data, the nIFR: 1.6% (CI 95%: 0.79%-2.8%), which is consistent with our more accurate estimate calculated using the truncated distribution for all ages.

Age Range	cIFR	cCFR	Hospitalisation-to-death Distribution

All ages combined	1.0% (0.35% - 2.5%)	2.1% (0.71% - 5.0%)	Non-truncated (Figure 1A)
	1.2% (0.39% - 2.7%)	2.3% (0.75% - 5.3%)	Truncated (Figure 1B)
70+	5.2% (1.9% - 11%)	10% (3.8% - 22%)	Non-truncated (Figure 1A)
	6.2% (2.3% - 13%)	12% (4.7% - 26%)	Truncated (Figure 1B)

Table S1: cIFR and cCFR estimates calculated using the reported case and death data on the Diamond Princess cruise ship [2]. Correction was performed using equation (1) and the hospitalisation-to-death distribution in [8].

Age Range	No. of passengers	Symp. cases	Asymp. cases	nCFR	Expected deaths using external nCFR	Observed deaths on cruise ship
0 - 9	16	0	1	0.0% (0.0% - 0.9%)	0 (0 - 0)	0
10 - 19	23	2	3	0.2% (0.0% - 1.0%)	0 (0 - 0)	0
20 - 29	347	25	3	0.2% (0.1% - 0.4%)	0.05 (0.02 - 0.10)	0

30 - 39	428	27	7	0.2% (0.1% - 0.4%)	0.06 (0.04 - 0.10)	0
40 - 49	334	19	8	0.4% (0.3% - 0.6%)	0.08 (0.06 - 0.12)	0
50 - 59	398	28	31	1.3% (1.1% - 1.5%)	0.36 (0.31 - 0.43)	0
60 - 69	923	76	101	3.6% (3.2% - 4.0%)	2.74 (2.5 - 3.1)	0
70 - 79	1015	95	139	8.0% (7.2% - 8.9%)	7.6 (6.8 - 8.4)	3
80 - 89	216	29	25	14.8% (13.0% - 16.7%)	4.28 (3.8 - 4.9)	4
Totals	3711	301	318		15.15 (13.5 - 17.1)	7

Table S2: Age stratified data of symptomatic (symp.) and asymptomatic (asymp.) cases on-board the Diamond Princess [2], [3], along with the nCFR estimates given in [9], the expected number of cases in each age group if the nCFR estimates were correct where the total number of expected deaths under these estimates was 15.15 and age stratified observed/expected death ratios.

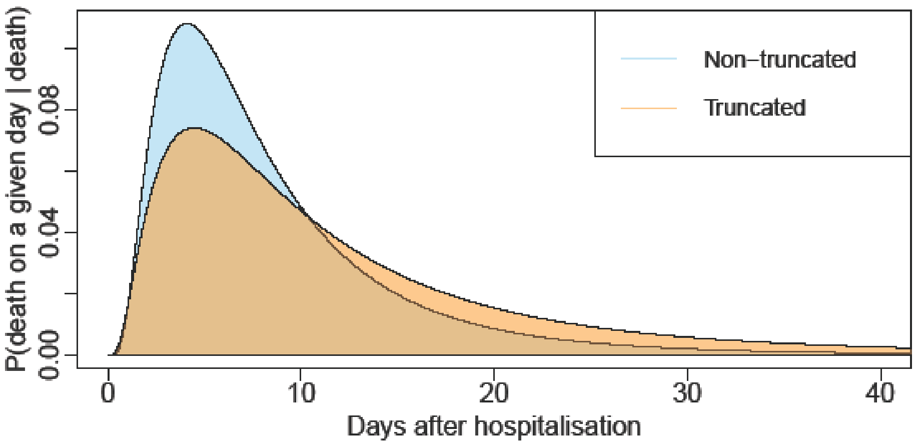

Non-truncated and truncated delay distributions

Figure S1: The time-to-death distributions and case and death data used to calculate the cCFR estimates. Panel A: the delay distributions of hospitalisation-to-death; both are a lognormal distributions fitted and reported in Linton et al.(Table 2) using data from the outbreak in Wuhan, China. The non-truncated distribution has a mean of 8.6 days and SD of 6.7 days; the right-truncated distribution has a mean of 13 days and SD of 12.7 days. Panels B and C: the case and death time series (respectively) of passengers on-board the ship.

References

1 Linton NM, Kobayashi T, Yang Y et al. Incubation period and other epidemiological characteristics of 2019 novel coronavirus infections with right truncation: A statistical analysis of publicly available case data. Journal of Clinical Medicine 2020;9:538.

2 National Institute of Infectious Diseases. Field Briefing: Diamond Princess COVID-19 Cases. https://www.niid.go.jp/niid/en/2019-ncov-e/9407-covid-dp-fe-01.html (accessed 3 Mar2020).

3 National Institute of Infectious Diseases. Field Briefing: Diamond Princess COVID-19 Cases. https://www.niid.go.jp/niid/en/2019-ncov-e/9417-covid-dp-fe-02.html (accessed 3 Mar2020).
